# Supplementary figures and images for: Adapterama II: universal amplicon sequencing on Illumina platforms (TaggiMatrix)
Source: PeerJ. 2019 Oct 11;7:e7786. doi: 10.7717/peerj.7786 (PMC6791344; doi:10.7717/peerj.7786)

# Index Positions in TaggiMatrix Complete Library

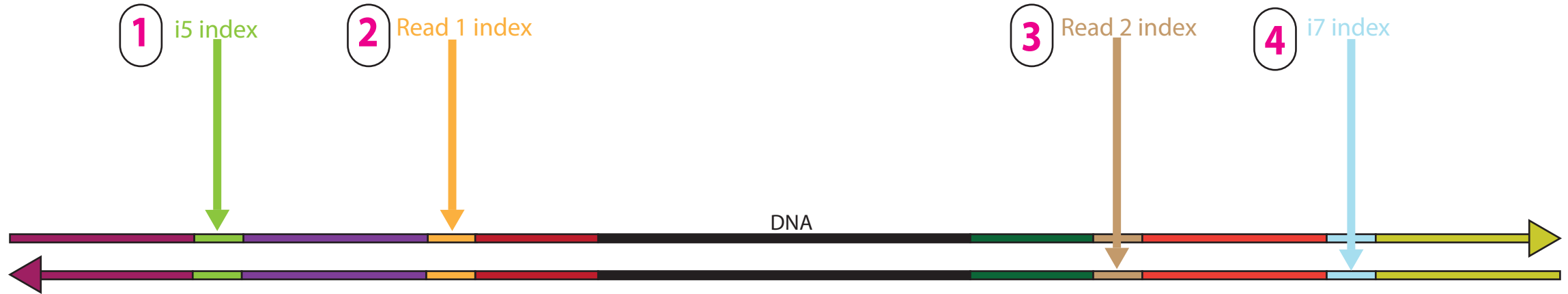

Supplement: Figure S1 — Double stranded amplicon library product after implementation of TaggiMatrix. Indication tags and indexes incorporated through the use of Fusion primers and iTru/iNext primers, respectively. [file peerj-07-7786-s001.pdf]

# TaggiMatrix Library Components

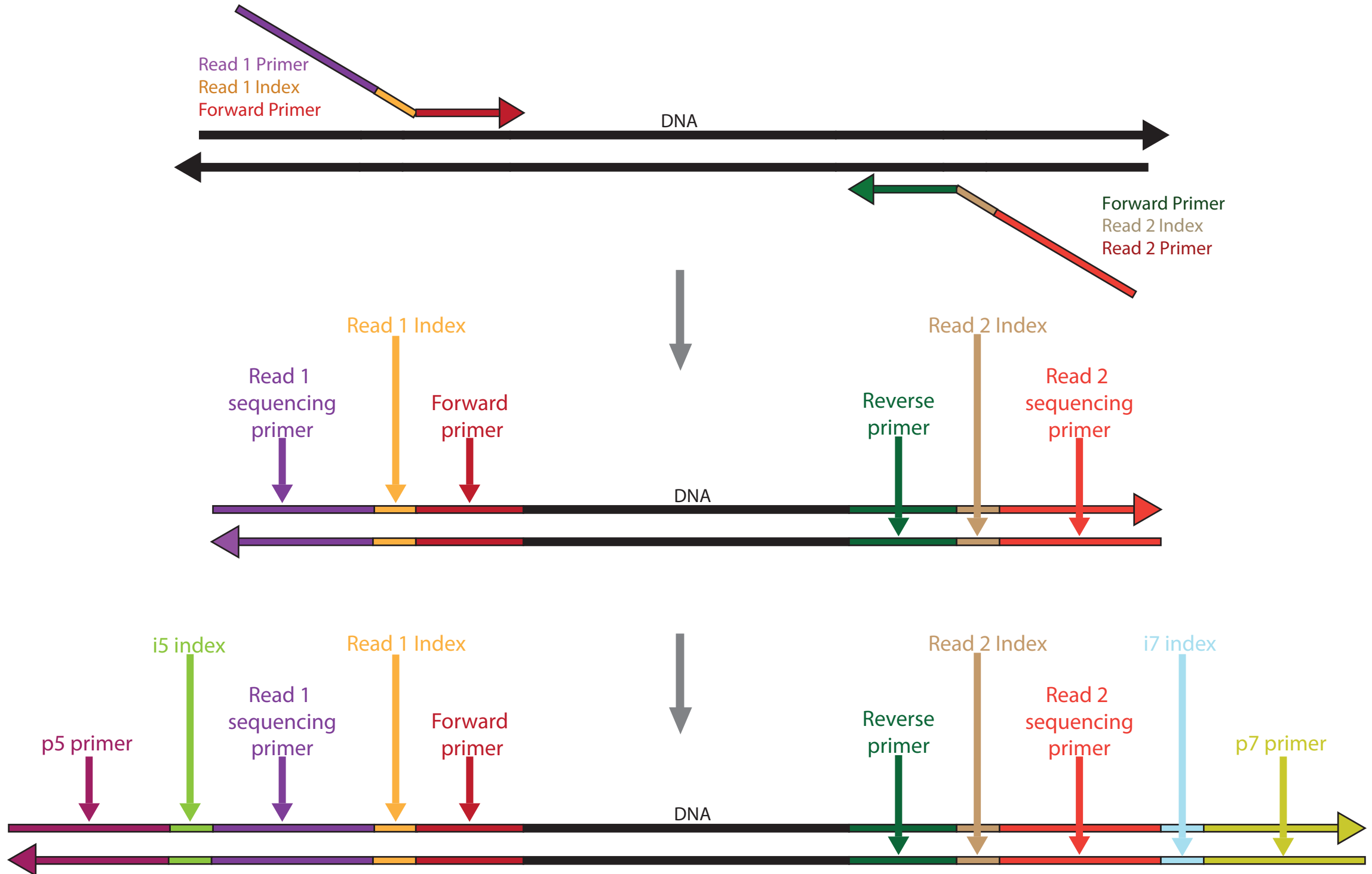

Supplement: Figure S2 — First, locus specific fusion primers with tags are used to amplify the target DNA region. From this step pooling is possible thanks to the presence of indexes. Then library amplification with the use of iTru universal primers with indexes that allows pool labeling and incorporation of Illumina platform oligos (P5 and P7). [file peerj-07-7786-s002.pdf]
